# SARS-CoV-2 Infection in Cancer Patients: Effects on Disease Outcomes and Patient Prognosis

**DOI:** 10.3390/cancers12113266

**Published:** 2020-11-05

**Authors:** Gaurav Seth, Saira Sethi, Shristi Bhattarai, Geetanjali Saini, Chandra Bhushan Singh, Ritu Aneja

**Affiliations:** 1 Department of Biology, College of Arts and Sciences, Georgia State University, Atlanta, GA 30303, USA; gseth1@student.gsu.edu (G.S.); sbhattarai1@student.gsu.edu (S.B.); sainigeetan@gmail.com (G.S.); 2 Maulana Azad Medical College and Associated Lok Nayak Hospital, New Delhi 110002, India; sairasethi94@gmail.com; 3 Department of Surgery, Maulana Azad Medical College and Associated Lok Nayak Hospital, New Delhi 110002, India; drchandrabhushansingh@gmail.com

**Keywords:** COVID-19, SARS-CoV-2, cancer prognosis, immunosuppression

## Abstract

**Simple Summary:**

Cancer patients are a highly vulnerable subgroup in this coronavirus disease 2019 (COVID-19) pandemic. This crisis has dramatically disrupted the continuous care provided to cancer patients, as well as diagnostic and therapeutic procedures. Thus, studies that help gain insights into COVID-19 prevalence, disease severity, prognosis, and clinical outcomes in cancer patients are of great importance. In this review, we outline the current knowledge of disease outcomes and prognoses for cancer patients with severe acute respiratory syndrome coronavirus 2 (SARS-CoV-2) and learn if the severity is exacerbated by cancer type, anticancer therapies, gender, behavioral risk factors (e.g., smoking, alcohol consumption), and comorbidities. Our article will help clinicians determine cases where treatment can be postponed during the pandemic and will encourage further research to better understand the impact of SARS-CoV-2 infection on cancer patients.

**Abstract:**

The severity of coronavirus disease 2019 (COVID-19) symptoms and outcomes vary immensely among patients. Predicting disease progression and managing disease symptoms is even more challenging in cancer patients with severe acute respiratory syndrome coronavirus 2 (SARS-CoV-2). Cancer therapies, including chemotherapy, radiotherapy, and immunotherapy, often suppress the immune system, rendering cancer patients more susceptible to SARS-CoV-2 infection and the development of severe complications. However, data on the effects of immunosuppression on COVID-19 outcomes in cancer patients remain limited. Further investigations are warranted to better understand the implications of SARS-CoV-2 infection in cancer patients, particularly those that are immunocompromised. In this review, we outline the current knowledge of the effects of SARS-CoV-2 infection in cancer patients.

## 1. Introduction

Severe acute respiratory syndrome coronavirus 2 (SARS-CoV-2) is the causative agent of the ongoing coronavirus disease 2019 (COVID-19) pandemic, which primarily affects the respiratory tract. Albeit highly contagious, the virus shows lower virulence than its precursors. With a reproductive rate of 2.68, it also exhibits a higher rate of transmission and infectivity [1,2]. Although SARS-CoV-2 shares a 79.5% sequence identity to SARS-CoV, emerging evidence shows that the case fatality rate of SARS-CoV-2 is considerably lower than those of SARS-CoV and MERS-CoV, which are 15% and 35%, respectively [3,4].

Although COVID-19 affects all age groups, children are often asymptomatic or present with a relatively milder disease course than adults [4]. Adults with underlying conditions, including diabetes, chronic obstructive pulmonary disease (COPD), and hypertension, as well as the elderly and immunocompromised patients, frequently require hospitalization, intensive care unit (ICU) admission, and respiratory interventions (e.g., oxygen therapy and mechanical ventilation/intubation). Additionally, these patient subgroups are more likely to develop severe, life-threatening complications, such as acute respiratory distress syndrome, coagulation dysfunction, and septic shock [2].

Cancer patients are a highly vulnerable subgroup in this COVID-19 crisis. Notably, the COVID-19 mortality rate is two times higher in cancer patients than the general population (5.6% vs. 2.3%) [1]. Cancer patients are also more susceptible to COVID-related complications, which may cause death [5]. As cancer patients frequently visit hospitals and clinics for therapy, they are more susceptible to viral infections, including COVID-19 [6]. In addition, many cancer patients are immunocompromised due to immunosuppressive chemotherapy, contributing to the higher severity of COVID-19 in cancer patients. Moreover, smoking history, which is strongly linked to cancer, has been found to increase the risk of ICU admission and the need for invasive ventilation in COVID-19 patients [7].

Given the risk of continuing immunosuppressive anticancer treatment and subsequent exposure to SARS-CoV-2 in hospitals, medical authorities have taken precautionary measures, such as discouraging hospital admission in clinically stable patients and adopting telemedicine to prevent nosocomial infection. Furthermore, following stringent distancing guidelines and using personal protection equipment (PPE) are strongly recommended to reduce the risk of exposure in immunocompromised cancer patients [8,9].

The ongoing COVID-19 pandemic has dramatically disrupted the continuous care provided to cancer patients, as well as diagnostic and therapeutic procedures, such as positron emission tomography scans, magnetic resonance imaging, mammograms, biopsies, elective surgeries, chemotherapy, and radiotherapy [10]. Hence, COVID-19 hinders cancer diagnosis and treatment, thereby imposing an additional burden on the already overwhelmed healthcare system. There needs to be an assessment of the risks that arise due to these delays in clinical treatment and their effects on the survival of cancer patients. Our study covers various cancer types and analyzes complex cases to decipher the effect of immunosuppression, comorbidities, gender, anticancer therapies, and behavioral risk factors on cancer patients infected with SARS-CoV-2. This multidimensional study will provide interesting insights into COVID-19 prevalence, disease severity, prognosis, and clinical outcomes in cancer patients. Such detailed analysis may help healthcare facilities determine cases where treatment can be postponed in the wake of the COVID-19 pandemic. This is essential in order to address the great uncertainty in the field of cancer care in the midst of the pandemic. This review was carried out by systematically searching databases like PUBMED, Google Scholar, and EMBASE. The greater part of the data was collected from case series and uncontrolled studies. All studies of COVID-19 patients or those with typical symptoms (even if untested or tested with negative results) and malignancy (with or without comorbidity) have been included in our analysis.

## 2. Virus Replication, Pathogenesis, and Disease Pathology

SARS-CoV-2 enters lung epithelial cells using angiotensin-converting enzyme 2 (ACE2) as its entry receptor. After the binding of the virus to ACE2, viral RNA enters the cell nucleus and undergoes replication. Cleavage of SARS-CoV-2 spike (S) protein by the host transmembrane protease serine 2 (TMPRSS2) facilitates the production and maturation of viral proteins, which are then released [11]. In humans, the SARS-CoV-2 S protein binds ACE2 with a higher affinity than the SARS-CoV S protein, contributing to the high transmission rate of SARS-CoV-2 [12]. The most common symptoms of COVID-19 are fever, dyspnea, cough, malaise, and diarrhea, with an estimated incubation period of 1–14 days [13].

Extensive replication of SARS-CoV-2 in the cells of the pulmonary epithelium is the primary cause of respiratory tract symptoms. The virus can also replicate in non-pulmonary cell lines, including Huh7 (hepatic) and 293T (renal) cells. Consistently, approximately 40% of COVID-19 patients develop hepatic dysfunction, and up to 7% exhibit acute kidney injury. To some extent, SARS-CoV-2 can also replicate in U251 (neuronal) cells, which can explain the neurological symptoms of COVID-19, such as confusion, anosmia, and ageusia [13].

Some COVID-19 patients develop cytokine storm syndrome [14]. High viral titers induce the release of proinflammatory cytokines, such as interleukin (IL)-6, IL-8, and IL-1β. These cytokines cause immunopathogenic damage to pulmonary and interstitial tissues and may induce apoptosis in lung endothelial and epithelial cells. Lung tissue damage eventually leads to vascular leakage and alveolar edema, ultimately resulting in hypoxia [15,16]. Thus, a cytokine storm is considered a leading pathogenic factor, causing lung injury, acute respiratory distress syndrome, and multiple organ failure [15,17]. The immune responses initiated to inhibit viral replication may exacerbate COVID-19 symptoms and cause death.

The pathological changes associated with SARS-CoV-2 infection are becoming increasingly evident. Sufang Tian et al. reported that two lung cancer patients with a history of hypertension and diabetes, who underwent unilateral pneumonectomy before COVID-19 diagnosis, were initially asymptomatic with clear bilateral respiratory auscultation. However, after the operation, the patients showed dyspnea, chest tightness, and decreased oxygen saturation. Their computed tomography (CT) scans indicated the presence of characteristic ground-glass opacities. Histological examination of the resected lungs revealed edema and diffuse interstitial thickening of the alveolus, proteinaceous and fibrin exudates, focal reactive hyperplasia of type 2 pneumocytes, and multinucleated giant cells [18].

ACE converts angiotensin I to angiotensin II, which, upon binding to AT1 receptors, stimulates the production of proangiogenic factors, thereby promoting cell proliferation in breast and lung cancers [19,20]. During cell entry, SARS-CoV-2 binds to and inhibits ACE2, reducing the levels of functional ACE2 [20,21,22,23,24,25]. ACE2 inhibition hampers the conversion of angiotensin II to angiotensin 1–7, which is known to have antiproliferative and anti-inflammatory effects [20,22,23,24,25]. Elevated levels of angiotensin II may also increase angiotensin III levels, further promoting tumorigenesis and angiogenesis [20]. Hence, SARS-CoV-2 infection may promote cancer development and progression. Moreover, Kuba et al. demonstrated that SARS-CoV S-protein-mediated ACE2 downregulation contributes to acute lung failure since ACE2 plays a protective role in acute lung injury. ACE2 downregulation upon SARS-CoV infection diminishes the protective role of ACE2 in lung pathologies [26]. Although a similar molecular link remains to be demonstrated for SARS-CoV-2, the close homology between their S proteins portends a similar worsening of lung pathologies and can prove to be fatal for lung cancer patients.

## 3. Immunosuppression in Cancer Patients

Immunosuppressive antitumor drugs impair humoral immunity and neutrophil function, increasing the risk of viral infection [27]. Notably, immunosuppressed individuals are more prone to influenza infections than immunocompetent individuals and exhibit prolonged virus shedding; they are more likely to develop severe complications that require intensive care [28]. Hence, routine influenza vaccination is recommended for immunocompromised individuals [29].

Studies of the 2009 H1N1 pandemic revealed that while most influenza infections caused lower respiratory tract symptoms, the need for hospitalization was more common in patients with solid tumors and hematologic malignancies undergoing chemotherapy [30]. An influenza outbreak occurred among 19 hospitalized cancer patients, 13 of whom were nosocomial infection cases. These patients were undergoing chemotherapy and had laboratory-confirmed neutropenia. Fourteen patients were diagnosed with upper respiratory tract illness, one patient had bronchiolitis, and four had atypical viral pneumonia (two of them eventually died) [31]. Immunocompromised individuals may also act as carriers for drug-resistant viruses, acting as potential sources of community-spread of drug-resistant viral strains [28].

Cancer patients who receive certain anticancer treatments have suppressed immune responses and are more likely to develop SARS-CoV-2-associated complications [32,33]. Immune system dysfunction, including overexpression of immunosuppressive cytokines, impaired maturation of dendritic cells, and elevated numbers of immunosuppressive leukocytes, can lead to cancer development and progression [32]. However, immunosuppression alone may not increase the risk of severe pulmonary disease. In immunocompromised patients, tobacco consumption history has been found to increase the risk of COPD, which is a risk factor for severe COVID-19 [27,34]. The increased ACE2 levels in individuals with a smoking history may also contribute to their increased susceptibility to SARS-CoV-2 infection [34,35]. Anemia and hypoproteinemia due to nutritional deterioration in cancer patients may also affect their immunocompetence and increase susceptibility to infectious respiratory pathogens [36].

Dai and colleagues found that patients with hematologic cancers, such as leukemia, lymphoma, and myeloma, were more immunocompromised and had a higher mortality rate than patients with solid tumors [37,38]. In line with these findings, a recent study has shown that patients with hematologic cancers, especially those with leukemia, were more susceptible to SARS-CoV-2 infections than patients with solid cancers. Patients with hematologic cancers were also found to have a higher risk of severe outcomes and mortality after adjusting for age and sex. Moreover, the likelihood of complications requiring ICU admission or supplemental ventilation was found to be higher in patients with a recent history of anticancer chemotherapy. In combination with myelosuppressive treatments, immunological dysfunction enables the replication of SARS-CoV-2 in the host’s body, which may lead to multiorgan failure and a cytokine storm [39].

On the other hand, diminished cytokine activity and immunosuppression in cancer patients may be beneficial in the context of SARS-CoV-2 infection. Spezzani et al. reported that in a COVID-19 patient with stage 4 breast cancer, receiving immunosuppressive chemotherapy, leucopenia may have contributed to the rapid recovery and prevention of a cytokine storm and other COVID-19 complications [40].

It has also been reported that patients undergoing chemotherapy and those with COVID-19 exhibit similar clinical characteristics, complicating the diagnosis of COVID-19 in patients receiving chemotherapy. Kobayashi et al. reported that a 49-year-old woman undergoing chemotherapy for serous ovarian cancer and peritoneal and lymph node metastasis had typical CT findings of COVID-19, such as bilateral peripheral ground-glass opacities; however, the patient tested negative for the disease by RT-PCR. This finding suggests that it is necessary to differentiate the complications due to chemotherapy from those caused by SARS-CoV-2 infection [41].

Table 1 summarizes the findings of 26 studies assessing the clinical outcomes and characteristics of SARS-CoV-2-infected cancer patients. Some of these studies assessed a single outcome (e.g., mortality or ICU admission), whereas others investigated numerous outcomes.

## 4. Effect of Comorbidities on Immune Status and COVID-19 Outcomes

In a study of hospitalized SARS-CoV-2 patients, Yifan Meng et al. found that patients with a history of cancer had worse prognosis and a higher mortality rate (29.4%) than those without cancer (10.2%) [38]. This higher risk might have resulted from post surgery effects, a weakened immune system due to anticancer therapies, and inflammation in the tumor microenvironment [38]. Additionally, cancer patients exhibited elevated levels of C-reactive protein, ferritin, procalcitonin, IL-2, and IL-6, as well as an increased erythrocyte sedimentation rate, indicative of a proinflammatory state [38].

Old age is another significant risk factor for severe COVID-19 [42]. The mortality rate of COVID-19 in cancer patients aged between 70 and 79 years is 8%, whereas patients in their eighties exhibit a mortality rate of 15% [43]. Among a cohort of 52 cancer patients with a median age of 63 years, Yang et al. reported that 19 patients were critically ill, and 11 died from complications (mortality rate of 21.2%). Patients afflicted with additional comorbidities, such as diabetes, hypertension, coronary artery disease, and respiratory conditions, accounted for 63.5% of the cohort [44]. In another single-center study, Yang et al. analyzed 52 severely ill COVID-19 patients admitted to the ICU. Of these patients, 50% had chronic comorbidities. Two patients had malignancies; one of these patients recovered, and the other died [45]. The adverse events associated with COVID-19 could possibly be overestimated due to an underreporting of these comorbidities. Regardless, patients with underlying conditions should be carefully monitored [46].

However, the presence of comorbidities does not necessarily lead to severe complications. In a study of 27 breast cancer patients with underlying conditions (hypertension in 15 patients, diabetes in 6, and pulmonary diseases in 6), Kalinsky et al. reported that 74% of the patients did not require hospitalization, and none of the patients required invasive ventilation. However, five patients required oxygen therapy. Follow-up of these patients showed that only one patient, an 87-year-old male patient with multiple comorbidities and a history of smoking, died due to complications [47].

In a study of four COVID-19 patients with cancer and additional comorbidities (e.g., diabetes and hypertension), Wang et al. found a significantly higher risk of complications in patients with at least two comorbidities than those with one or no comorbidities [48]. Consistently, in a large study of 1590 patients (of whom 399 had at least one underlying condition), Guan et al. found a higher hazard ratio in patients with comorbidities [46]. Notably, a recent study reported that patients with metastatic cancers had the highest COVID-19 mortality rate [49]. Future studies are required to determine whether the stage of cancer is an independent risk factor for severe COVID-19.

## 5. Effect of Immunosuppressive Drugs on COVID-19 Severity

Mounting evidence suggests that immunocompromised cancer patients, especially those recently treated with anticancer agents, may have higher COVID-19 morbidity and mortality rates. Zhang et al. examined 28 cancer patients, 7 of whom had lung cancer and had received anticancer treatment 14 days before SARS-CoV-2 infection. Fifteen patients developed severe illness, and eight of them died [1]. Similarly, Yang et al. studied 52 cancer patients (lung, breast, colorectal, thyroid, and cervix cancers) who tested positive for SARS-CoV-2, 10 of whom had received chemotherapy or immunotherapy and surgical resection the month before the infection. Eleven patients died of complications (mortality rate, 21.2%), and 19 patients were critically ill [44].

Immune dysregulation and ACE2 overexpression are common among older individuals [50], possibly contributing to their higher risk of adverse COVID-19 outcomes [51,52]. Lescure et al. described an 80-year-old patient who presented with fever and diarrhea and a history of thyroid cancer. The disease progressed rapidly, and the patient experienced multiorgan failure, acute respiratory distress syndrome (ARDS), sepsis, and acute kidney injury. Despite treatment with remdesivir and antibacterial agents, the patient died 24 days after hospitalization [53]. Serum cytokine levels are significantly elevated in patients with ARDS and are correlated with the fatality rate [15].

Although we should not underestimate the risk of severe COVID-19 in cancer patients, some cancer patients only develop mild symptoms. Spezzani et al. described a breast cancer patient treated with mastectomy, radiotherapy, and chemotherapy, who later presented with lymph node and bone metastases. The patient received antineoplastic chemotherapy with fulvestrant and abemaciclib, which was suspended after the patient developed mild COVID-19 symptoms [40]. Hong Jin et al. reported a 39-year-old patient diagnosed with COVID-19 after presenting with fever and dyspnea. Although the patient was previously treated with R-CHOP chemotherapy due to chronic lymphocytic and non-Hodgkins leukemia, supplemental oxygen alleviated the respiratory symptoms [54].

Due to immune suppression, patients undergoing antitumor treatments may not develop COVID-19 symptoms. Tian et al. examined two asymptomatic patients who underwent lobectomy for lung cancer. The first patient was a 73-year-old hypertensive man who developed symptoms during postoperative care but responded well to treatment. The second patient was an 84-year-old woman with hypertension and diabetes who developed dyspnea after lobectomy and tested positive for SARS-CoV-2. Despite treatment with antibiotics and supplemental oxygen, the patient died 29 days after lobectomy [18].

Cancer patients are often administered corticosteroids as part of their treatment regimen and to manage disease symptoms (e.g., to suppress lung inflammation). COVID-19 is associated with elevated inflammation, and resultant symptoms (e.g., fever, muscle ache) are early signs of possible SARS-CoV-2 infection. Further, pathological immune profiling can yield preliminary information about an active infection in the body. However, in cancer patients receiving corticosteroids, such early indicators of concomitant SARS-CoV-2 infection may be masked due to immunosuppression. Hence, the prudent approach to monitor these patients is by regularly testing them for the presence of SARS-CoV-2 (by RT-PCR or antigen testing) [7,55].

## 6. COVID-19 Progression in Cancer Patients

Numerous studies and case reports have addressed the course of COVID-19 in cancer patients. Some studies have reported that the presence of comorbidities, in addition to malignancy, further compromise the patient’s immune system. Cancer patients are often older, have dyspnea, and a smoking history, all of which are risk factors for severe COVID-19 [56]. In a study of nine COVID-19 patients, Zeng et al. reported that three patients developed severe symptoms; one of these patients died of multiorgan failure three days after the onset of symptoms. The median time from symptom onset to hospitalization was four days [57]. In another study, Hrusak et al. showed than among nine children with cancer who contracted SARS-CoV-2, none of them required ICU admission, suggesting that children undergoing cancer treatment may have a milder disease course, and, thus, the treatment should not be delayed (Table 2) [58].

It is too early to conclude whether the immunocompromised status of cancer patients affects the course of COVID-19, especially considering the conflicting reports. Some studies show that both young and elderly immunocompromised cancer patients may have a favorable disease course compared with the rest of the population with comorbidities, whereas others contend that cancer patients have worse prognosis and outcomes.

## 7. Effects of Cancer Therapies on COVID-19 Outcomes

Increased risk of severe complications and morbidity in COVID-19 patients seems to be associated with progressive cancer and a high Eastern Cooperative Oncology Group (ECOG) performance status (higher than 2) [35]. However, findings on COVID-19 outcomes in cancer patients are conflicting. In a large cohort study (*n* = 800 patients), a mortality rate of 27% was reported in 281 patients with active cancer who had received chemotherapy the month before contracting SARS-CoV-2. In contrast, the mortality rate of patients who did not receive chemotherapy was 29%; thus, the authors concluded that cytotoxic chemotherapy is not a significant risk factor for COVID-19 severity and mortality [49]. Similarly, patients receiving other anticancer therapies, such as immunotherapy (*n* = 44; odds ratio (OR), 0.59; 95% confidence interval (CI), 0.27–1.27; *p* = 0.177), hormonal therapy (*n* = 64; OR, 0.90; 95% CI, 0.49–1.68; *p* = 0. 744), radiotherapy (*n* = 76; OR, 0.65; 95% CI, 0.36–1.18; *p* = 0.159), and targeted therapies (*n* = 72; OR, 0.83; 95% CI, 0.45–1.54; *p* = 0.559) did not display increased risk of mortality after adjusting for age, gender, and comorbidities [49]. In another similar cohort study of 928 cancer patients, 366 (39%) patients received anticancer treatments within four weeks of COVID-19 diagnosis. It was noted that 160 (44%) patients underwent cytotoxic therapy (22 died), and 206 (56%) underwent noncytotoxic therapy (23 died) [35]. The findings of these two studies suggest that anticancer treatment regimens do not influence the fatality rate of COVID-19. Nevertheless, the effects of myelosuppression or immune activation due to cytotoxic chemotherapy and immunotherapy, respectively, on COVID-19 outcomes should be examined in greater depth [35].

Immune checkpoint inhibitors (ICIs) have emerged as promising cancer therapies, exerting potent antitumor effects by unleashing immune responses. However, the influence of ICIs on the course of COVID-19 remains undetermined. Even though immune modulation may provide an advantage in eliminating SARS-CoV-2, it could also intensify the cytokine storm [59] and thereby promote ARDS [60] and severe pneumonitis [59]. Notably, treatment with ICIs was found to be a predictor of severe respiratory compromise, independent of age, type of cancer, and presence of other comorbidities [60].

## 8. Administration of Cancer Treatments during the COVID-19 Pandemic

Considering the heterogeneity of COVID-19 severity and outcomes, the administration of cancer treatments during the pandemic should be evaluated on a case-by-case basis. The implementation of appropriate preventive measures by hospitals and clinics would allow the continuation of oncological treatments. Di Giacomo et al. described a 74-year-old man with metastatic cutaneous melanoma receiving his 83rd anti-PD-1 monoclonal antibody treatment, who presented with fever, dyspnea, and oxygen saturation (SpO_2_) <94%, and eventually tested positive for SARS-CoV-2. He resumed his anticancer therapy after his COVID-19 symptoms improved [61]. Similarly, a 51-year-old woman receiving her 11th treatment cycle for cutaneous melanoma tested positive for COVID-19 and recovered within 10 days [61]. Abruzzese et al. described a 26-year-old pregnant patient receiving dasatinib for chronic myeloproliferative leukemia (CML). After developing a fever, her symptoms were managed symptomatically; she continued her CML treatment after recuperation [62]. These reports suggest that the continuation of cancer therapies after recovery from COVID-19 is safe.

Most elective surgeries have been postponed as excessive strain on healthcare systems due to COVID-19 has led to a decline in the quality of care provided to cancer patients [63]. The overwhelming requirement for ventilators in ICUs to treat critically ill COVID-19 patients poses an additional burden on the already exhausted medical community, raising the need for postponing surgery in stable cancer patients [64]. In a 65-year-old female COVID-19 patient with advanced ovarian cancer and peritoneum and liver metastasis, chemotherapy was continued for two cycles after COVID-19 diagnosis, although debulking surgery was postponed [41]. In cases where surgery is urgently required, the operation must be conducted in a negative-pressure operation theater to prevent aerosolization and viral transmission [65]. Huang et al. described a male COVID-19 patient who presented with obstipation and lower abdominal pain. CT scan findings indicated the presence of a colonic mass, causing bowel dilatation and intestinal obstruction. The patient underwent emergency surgery in a negative-pressure operation theater. The patient tested positive for SARS-CoV-2 during the postoperative period but recovered fully [66].

Lung cancer patients and those receiving antineoplastic treatment are more prone to life-threatening complications and rapid COVID-19 progression [1]. However, Zang and Huang described a 57-year-old hospitalized lung cancer patient who developed pyrexia and dyspnea and tested positive for SARS-CoV-2. The virus was eliminated after treatment with ritonavir/lopinavir [67], demonstrating that not all lung cancer patients have unfavorable COVID-19 outcomes. Therefore, cancer patients diagnosed with COVID-19 during curative treatment often recover fully, and cancer treatments can be safely continued.

## 9. Effect of Gender on COVID-19 Severity in Cancer Patients

Compared with men, women tend to have a milder COVID-19 course. Toll-like receptors (TLRs) and other immune-related genes present on the X chromosome are involved in the detection of single-stranded RNA viruses such as SARS-CoV-2. Innate immune cells are more active in women, rendering the phagocytosis and elimination of infected cells more effective [68]. Estrogen is also known to upregulate alpha-estrogen receptors in cytotoxic T-lymphocytes, increasing the production of interferons [19]. Thus, viruses elicit stronger cytotoxic immune responses in women, possibly contributing to the milder COVID-19 course [19].

Furthermore, women with breast cancer may have favorable COVID-19 outcomes. Kalinsky et al. described 27 COVID-19 patients (median age of 56 years) with a history of breast cancer (most with stage 1–3 breast cancer); 74% of patients did not require hospitalization, none of them required invasive ventilation, and 5 of them required oxygen therapy [47]. Vaugnat et al. examined 76 patients with active breast cancer, 59 of whom were diagnosed with COVID-19. Among breast cancer patients diagnosed with COVID-19, 39 were hormone receptor (HR)-positive, 10 were HER2/neu-positive, and the remaining 10 had triple-negative breast cancer. Additional comorbidities, such as hypertension and diabetes, were reported in 36% of patients. Hospitalization was required for 28 patients, and none of them received antiviral agents or immunomodulators; 76% of the patients recovered, and 7% died [69]. Although the findings of these studies demonstrate that breast cancer patients might have favorable COVID-19 outcomes, immunosuppressed patients should be closely monitored.

Tamoxifen, used to treat ER+ breast tumors, has antiestrogenic effects, thereby suppressing any protective effects of estrogen. Furthermore, tamoxifen may induce apoptosis in ER-expressing cells in lung tissues. By inhibiting P-glycoprotein 1, tamoxifen also suppresses the release of interferon from T-lymphocytes. Thus, breast cancer patients receiving tamoxifen may be at high risk of developing severe COVID-19 [19].

Mounting evidence suggests a higher COVID-19 burden in men than in women [68,70,71,72]. This gender disparity in terms of COVID-19 incidence and mortality was observed early during the pandemic in China, where the COVID-19 fatality rate was 2.8% in men and 1.7% in women [70]. Epidemiological case studies from Italy confirmed the higher SARS-CoV-2 infection rates in men [71]. Male cancer patients were found to be 79% more likely to contract SARS-CoV-2 than women [73]. Men express higher levels of ACE2, the entry receptor for SARS-CoV-2 [68,74]. Men are also more likely to smoke or have comorbidities, such as hypertension, diabetes, and obesity [68]. Active smoking further increases ACE2 expression levels in the lungs, facilitating the binding of SARS-CoV-2 to alveolar cells [35,68]. In addition, smoking alters the androgen-to-estrogen ratio, enhancing TMPRSS2 expression and facilitating the replication of SARS-CoV-2 [68]. Various studies have shown that men have higher levels of immune mediators linked to poor COVID-19 outcomes, including TNFSF13B, CCL23, and IL-16 [75]. Although estrogen is considered to augment humoral immune responses, testosterone seems to do the opposite, possibly contributing to the lower antibody titers seen in men [68].

Prostate cancer and COVID-19 share many risk factors, inducing age (>50), gender (male), comorbidities, behavioral factors (alcohol and tobacco use) [76], and high TMPRSS2 expression [77]. Androgen signaling regulates the expression of ACE2 [74] and TMPRSS2 [78], prominently observed in urogenital organs (prostate and testes) and the lungs [35,68]. The combination of immunosuppressive and oncogenic effects of androgen signaling dysregulation in prostate cancer patients may render them more susceptible to SARS-CoV-2 [68].

In a cohort of 9648 COVID-19 patients, 2.2% of patients had prostate cancer. The mortality rate in these patients was 23.7%, considerably higher than that in male patients with other malignancies (12.7%). Similarly, the rate of intubation was higher in patients with prostate cancer [68]. In the same study, prostate cancer patients receiving androgen deprivation therapy were four times less likely to contract SARS-CoV-2 [68]. In another study of 4532 patients, 9.4% had tumors. Among these patients, 118 had prostate cancer (2.6%), 66.1% of whom required hospitalization, and 15.3% died [73]. Together, these findings suggest that men have a higher risk of severe COVID-19 than women, reflected in the fact that the prognosis of COVID-19 is more favorable in breast cancer patients than in prostate cancer patients.

## 10. Cancer Treatment Strategies during the COVID-19 Pandemic

The current standard of care for COVID-19 is symptom management and respiratory assistance. Emerging COVID-19 therapies include drugs that inhibit various steps of viral replication, such as hydroxychloroquine, remdesivir, and ritonavir/lopinavir [2], as well as immunomodulatory agents suppressing cytokine storms [2,51]. Corticosteroids (e.g., dexamethasone) have proved to be lifesaving for critically ill patients requiring supplemental oxygen or mechanical ventilation [79]. Extracorporeal membrane oxygenation (ECMO) is recommended for patients with hypoxemia refractory to oxygen therapy [2,51]. Convalescent plasma therapy (anti-SARS-CoV-19 immunoglobulin-containing plasma from recovered patients) has been suggested to neutralize the virus, conferring passive immunity [51,80].

For cancer patients, efforts have been made to minimize the number of hospital visits and the extent of immunosuppression [81,82]. Although surgery is the treatment of choice in breast cancer, it is considered riskier than chemotherapy during the COVID-19 pandemic [64]. A hypofractionated regimen is recommended for these patients in order to reduce the number of hospital visits [83]. Nonoperative treatment for non-small cell lung carcinoma (NSCLC) involves concurrent or sequential chemo-radiotherapy, and surgery should be avoided in patients with COVID-19. If hemoptysis or tumor obstruction occurs, radiotherapy can be considered [84]. It has also been suggested that all lung cancer patients undergo routine COVID-19 testing to enable early detection, considering the overlap between lung cancer and COVID-19 symptoms [85].

For patients with hematological malignancies (e.g., multiple myeloma and malignant lymphoma), monthly infusions are recommended, and autologous stem cell transplantation may be continued in high-risk patients [86,87]. Hypofractionated chemo-radiotherapy is recommended for brain cancer patients to decrease the number of outpatient visits; biopsy and tumor resection are discouraged in patients with stable neurological symptoms [88]. Patients with tracheostomy or laryngectomy can transmit virus particles; hence, the use of closed-circuit ventilation, cuffed tracheostomy tubes with minimal manipulation, heat moisture exchange units, and adequate PPE is recommended [89]. Radical cystectomy and radical radiotherapy have similar outcomes in patients with urothelial cancers. The National Institute for Health and Care Excellence (NICE) guidelines recommend cisplatin-based chemotherapy after surgery to increase the survival rate. Neoadjuvant chemotherapy should be discouraged as it causes prolonged immunosuppression [90].

Due to their inherent immunosuppressive state and frequent visits to health centers, cancer patients are extremely susceptible to SARS-CoV-2 infections. A study of 12 COVID-19 patients with NSCLC showed that hospital exposure played an important role in their infection [91]. Teleconsultation allows medical professionals to evaluate patients remotely. Some hospitals have also implemented flexible protocols, allowing patients to receive outpatient therapy, delay dosing, or skip routine testing when necessary [92] to adapt to the rapidly changing social and political circumstances [65]. Adjuvant chemotherapy with curative intent may be continued in cancer patients while implementing extraordinary measures to prevent the transmission of SARS-CoV-2, as well as regular testing [92]. Furthermore, cancer patients should be closely monitored for long-term COVID-19 effects, in addition to cancer progression monitoring.

## 11. Concluding Thoughts

In this observational review, we outline the various challenges faced by cancer patients amidst the COVID-19 pandemic, as well as the effects of COVID-19 on cancer treatment and outcomes. Mounting evidence suggests that immunocompromised cancer patients have a higher risk of developing severe symptoms upon SARS-CoV-2 infection compared with the general population. Although cancer treatments often cause immunosuppression, current evidence suggests that immunosuppressive therapies do not affect the COVID-19 fatality rate. Clinicians and cancer patients should take preventive measures to minimize the risk of exposure to SARS-CoV-2. Additionally, cancer patients diagnosed with COVID-19 should be closely monitored, regardless of the severity of their symptoms.

Due to the novelty of the disease, little is known on the association between COVID-19 and cancer. Additional studies are required to better understand the impact of SARS-CoV-2 infection on the already compromised health of cancer patients, as well as the effects of cancer treatments on the course of COVID-19.

## Figures and Tables

**Table 1:** Clinical outcomes and characteristics of severe acute respiratory syndrome coronavirus 2 (SARS-CoV-2)-infected cancer patients.

Study No.	Authors	No. of Patients	Cancer Type	Outcome	Country	Comments
1	Spezzani et al.	1	Metastatic breast cancer	Recovered	Italy	Received chemotherapy in the month before infection
2	Liang et al.	16	Lung, breast, thyroid, colorectal, and adrenal cancers and lymphoma	50% ICU and recovered; 50% outpatient recovery	China	4 patients receiving chemotherapy recovered with a mild course
3	Kalinsky et al.	27	Breast cancer and metastatic breast cancer	26 recovered and 1 died	USA	Comorbidities—HTN (*n* = 15); diabetes (*n* = 6); COPD (*n* = 6)
4	Lescure et al.	1	Thyroid	ICU admission, mortality	China	
5	Yang et al.	52	Lung, breast, rectal, colon, cervical, and thyroid	38 received oxygen and recovered; 3 recovered; 11 died	China	10 patients received antitumor treatment the month before infection
6	Zeng et al.	9		1 died	China	5 received antitumor therapy in the last year; 2 severe cases
7	Zhang et al.	28	Lung	20 recovered; 8 died	China	7 received antitumor therapy 2 weeks before infection
8	Yang et al.	2		1 recovered; 1 died	China	50% had comorbidities
9	Wang et al.	4		3 recovered; 1 ICU	China	
10	Guan, Liang, Zhao, et al.	18		3 died, 5 ICU, and 2 IMV	China	Hazard rate for one/two comorbidities = 1.79/2.59
11	Hong Jin et al.	1	Non-Hodgkin lymphoma, CLL	Recovered	China	6 courses of 21 days of R-CHOP chemotherapy in 2007
12	Vaugnat et al.	59	Breast	4 died	France	
13	Zhang et al.	1	Multiple myeloma	Recovered	China	High IL-6, Tocilizumab treatment
14	Hrusak et al.	9	Osteosarcoma, Hepatoblastoma, Rhabdoid tumor, Wilms’ tumor, ALL, and Ewing sarcoma	Recovered	25 countries	
15	Kobayashi et al.	3	Ovarian serous cancer	Recovered	Japan, Korea	Received chemotherapy
16	Tian et al.	2	Lung	1 recovered; 1 died	China	
17	Abruzzese et al.	1	CML	Recovered	Italy	
18	Di Giacomo et al.	1	Metastatic cutaneous melanoma, locally advanced cutaneous melanoma	Recovered	Italy	
19	Yu et al.	12	Non-small cell lung carcinoma	1 ICU; 3 died	China	
20	Huang et al.	1	Colon	Recovered	China	Hepatitis B; intestinal obstruction
21	Zang and Huang	1	Lung	Recovered	China	
22	Chakravarty et al.	114	Prostate	27 died	USA	
23	Dai et al.	105	Lung, breast, thyroid, gastrointestinal, and hematologic cancers	11.43% died; 19.05% ICU admission; 34.29% severe symptoms; 9.52% IMV	China	Received treatment within 40 days of diagnosis: surgery (*n* = 8), radiotherapy (*n* = 13), chemotherapy (*n* = 17), targeted therapy (*n* = 4), or immunotherapy (*n* = 6)
24	Lee et al.	800	Lung, breast, prostate, and melanoma, among others	226 died	UK	Received cancer treatment within 4 weeks of diagnosis: surgery (*n* = 29), radiotherapy (*n* = 76), chemotherapy (*n* = 281), targeted therapy (*n* = 72), hormone therapy (*n* = 64), or immunotherapy (*n* = 6)
25	Kuderer et al.	928	Breast, prostate, and thoracic, among others	121 died, 132 admitted to ICU, and 116 IMV	USA, Canada, and Spain	Received cancer treatment within 4 weeks of diagnosis: cytotoxic therapy (*n* = 160) or noncytotoxic therapy (*n* = 260)
26	Montopoli et al.	786	Prostate, kidney/bladder, colorectal, lymphoma	292 hospitalized, 75 died	Italy	4 patients received androgen deprivation therapy

HTN, hypertension; CAD, coronary artery disease; ds, disease; COPD, chronic obstructive pulmonary disease; IMV, invasive mechanical ventilation; ICU, intensive care unit admission.

**Table 2:** The course of coronavirus disease 2019 (COVID-19) in 9 children with cancer.

Patient No.	Cancer Diagnosis	COVID-19 Symptoms	Previous Anticancer Treatment	Treatment for COVID-19	Outcomes
1	Osteosarcoma	Fever	Radiotherapy	Symptomatic management	Recovered
2	Hepatoblastoma	Fever, neutropenia	Chemotherapy	Azithromycin, G-CSF	Recovered
3	Rhabdoid tumor of cervix	Fever, neutropenia	Chemotherapy	Oxygen therapy, azithromycin, G-CSF	Recovered
4	Hepatoblastoma	Cough	Cisplatin-based chemotherapy	Symptomatic management	Recovered
5	Metastatic Ewing sarcoma	Fever, neutropenia	Chemotherapy	Lopinavir, ritonavir, hydroxychloroquine	Recovered
6	Wilms tumor	Fever, diarrhea, lymphopenia	Chemotherapy	Hydroxychloroquine	Recovered
7	Acute lymphoblastic leukemia	Fever, neutropenia	Chemotherapy	Symptomatic management	Recovered
8	Acute lymphoblastic leukemia	Fever, neutropenia	-	-	Recovered
